# Supplementary figures and images for: A potent and selective reaction hijacking inhibitor of Plasmodium falciparum tyrosine tRNA synthetase exhibits single dose oral efficacy in vivo
Source: PLoS Pathog. 2024 Dec 9;20(12):e1012429. doi: 10.1371/journal.ppat.1012429 (PMC11671014; doi:10.1371/journal.ppat.1012429)

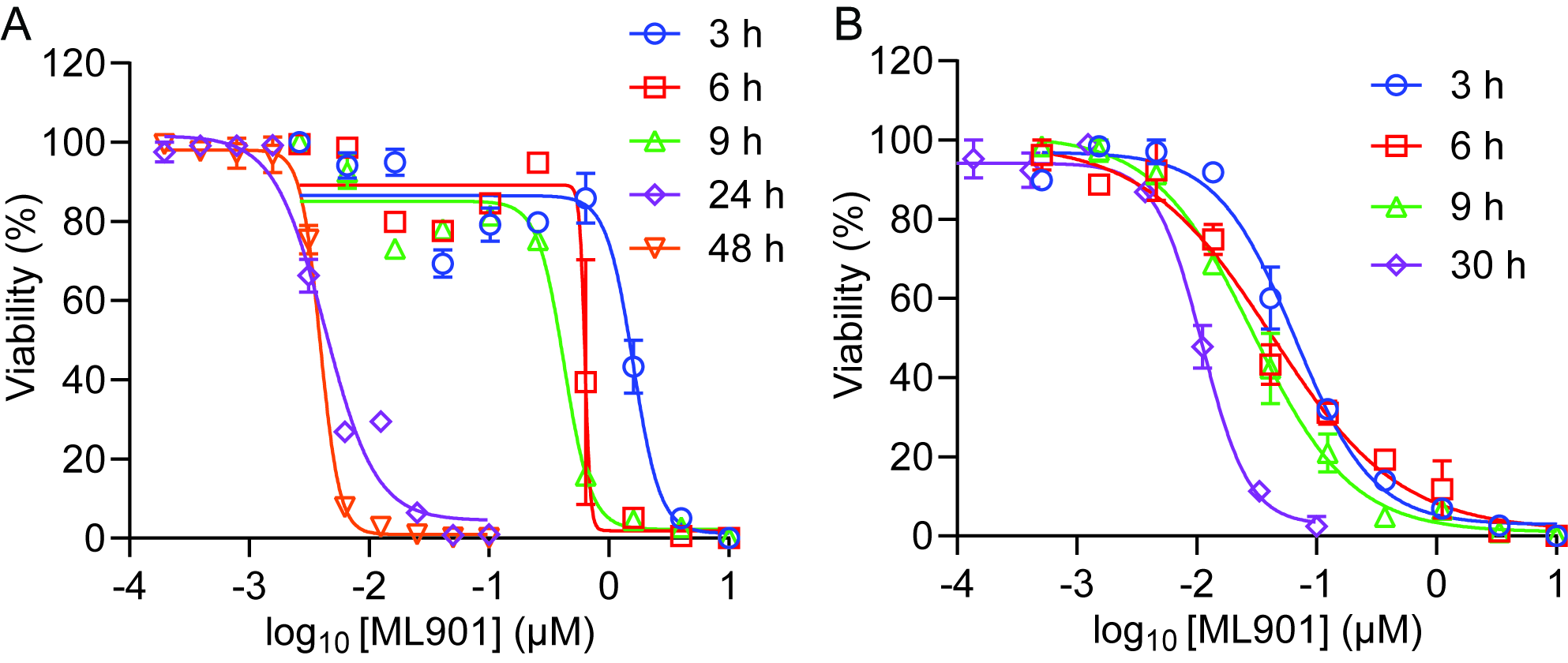

Supplement: S1 Fig — (A,B) A tightly synchronized culture of CAM 3.II Rev parasites (>70% of parasites within a 5-h time window) was subjected to pulses of ML901 for 3 h, 6 h, 9 h, 24 h, or continued exposure for 48 h (A) or 30 h (B), initiated at (A) trophozoite (25–30 h.p.i.) and (B) schizont (43–48 h.p.i.) stages. Flow cytometric analysis of Syto-61-labelled parasites in the cycle after the initiation of treatment assessed cell viability. Data are representative of three (trophozoite) and two (schizont) independent experiments. LD50 values are shown in Table S1. (TIF) [file ppat.1012429.s001.tif]

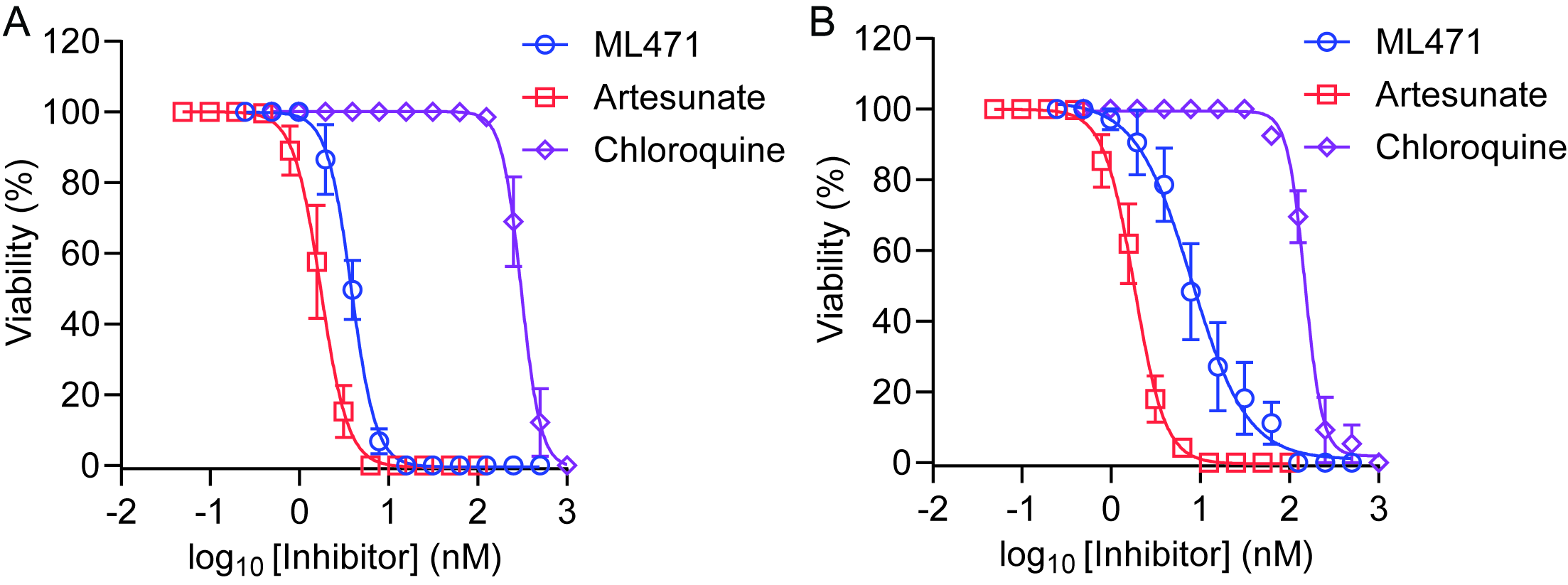

Supplement: S2 Fig — Compounds were assayed on P falciparum (A) isolates and P. vivax (B) Brazilian isolates collected from mono-infected patients. Data represent mean ± SEM. Median EC50 (nM) values, the range of values, and the numbers of isolates are shown in Table S2. (TIF) [file ppat.1012429.s002.tif]

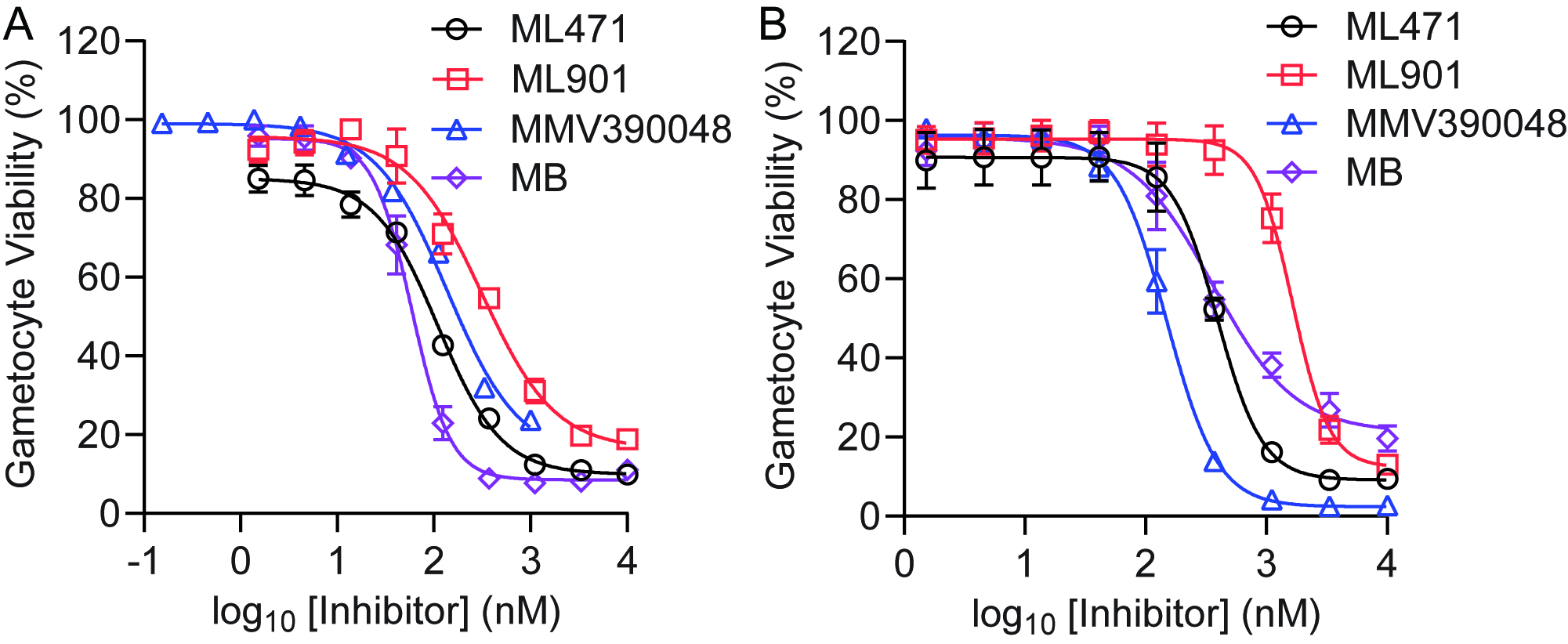

Supplement: S3 Fig — Gametocytocidal activity of the compounds was assessed against immature (>90% stage II/III) and mature (>95% stage V) stage gametocytes. MMV390048 and MB were used as controls. Data represent mean ± SEM from three independent experiments. Mean IC50 ± SEM values from the three independent experiments are presented in S3 Table. (TIF) [file ppat.1012429.s003.tif]

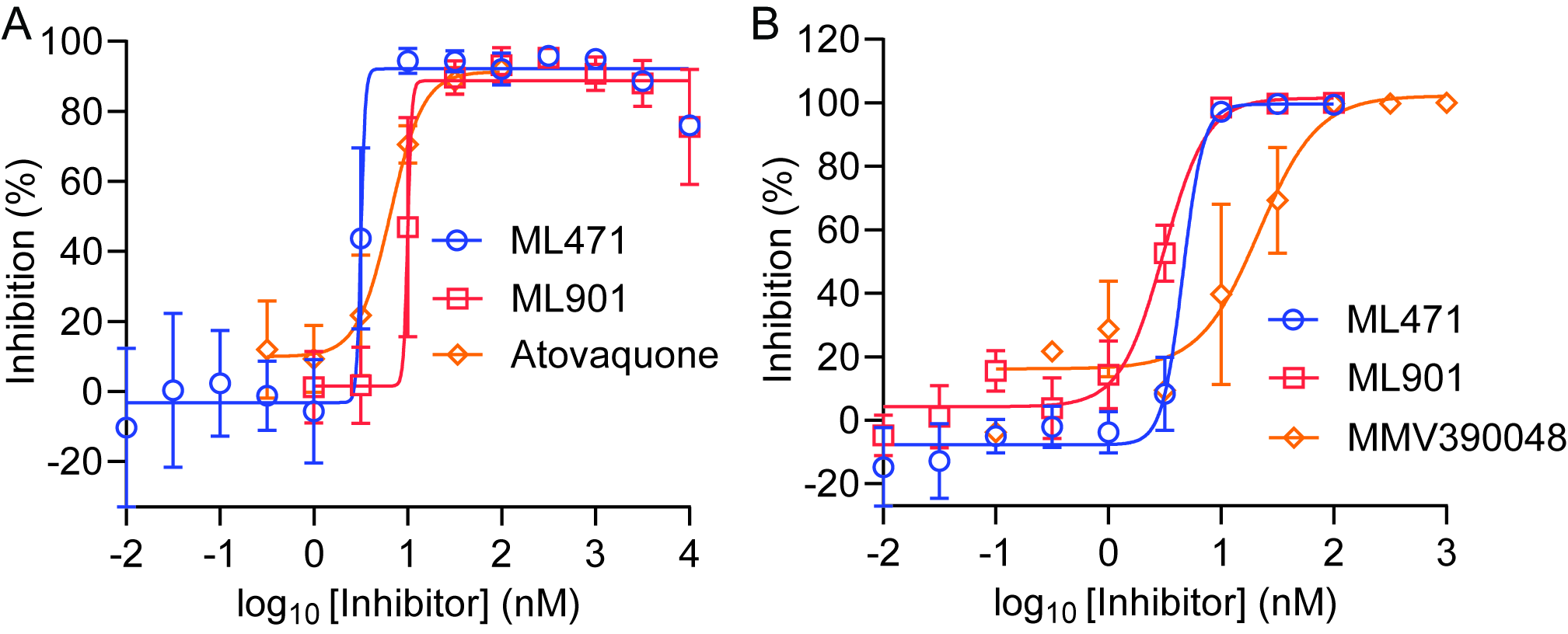

Supplement: S4 Fig — Human primary hepatocytes were infected with P. falciparum NF175 or NF135 sporozoites and cultured for four days. Anti-HSP70 was used to detect parasites in fixed cells using high content imaging. Atovaquone and MMV390048 were used as control compounds against NF175 and NF135 schizonts, respectively. Data values represent mean ± SEM from three independent experiments. IC50 values are shown in S4 Table. (TIF) [file ppat.1012429.s004.tif]

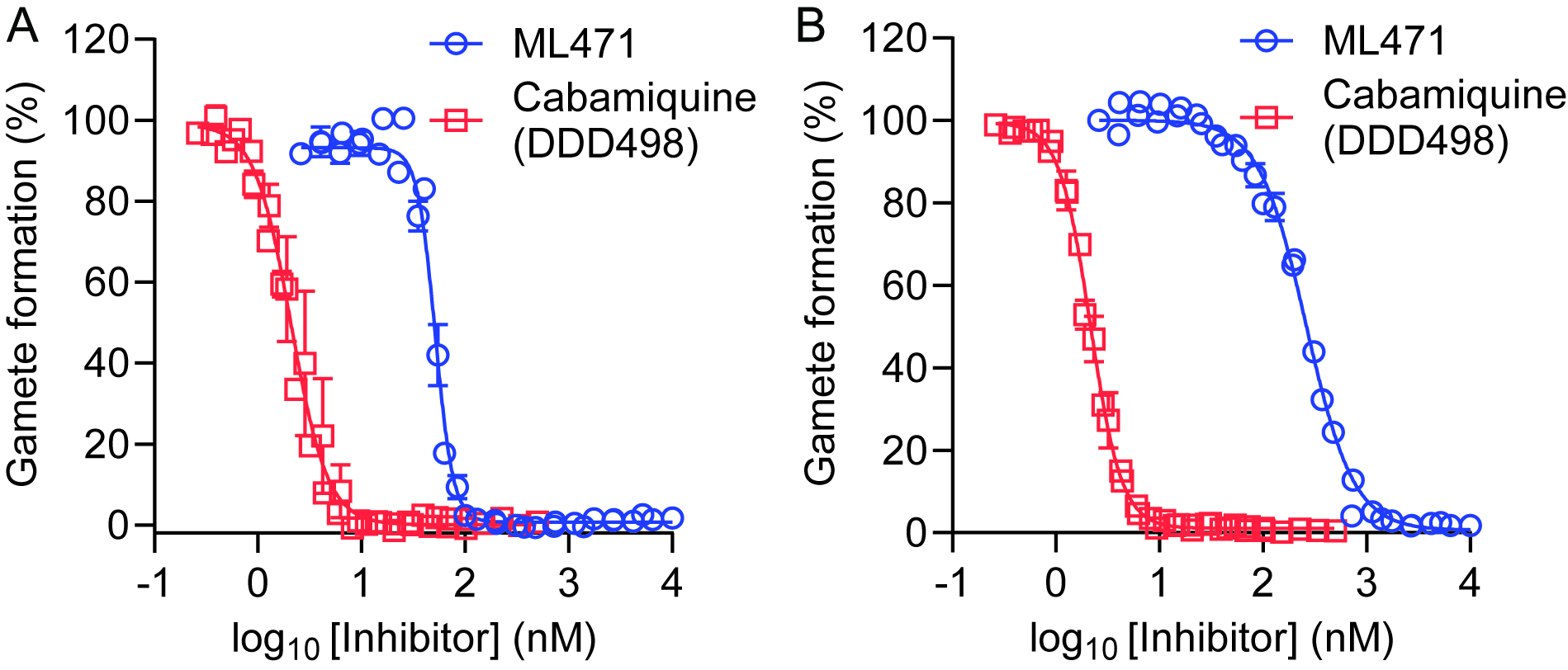

Supplement: S5 Fig — Inhibition of male (A) and female (B) gamete formation was assessed in the P. falciparum Dual Gamete Formation Assay. Cabamiquine (DDD498) and DMSO were used as positive and vehicle controls, respectively. Data represent mean ± SEM from 4–5 independent experiments. IC50 values are shown in S5 Table. (TIF) [file ppat.1012429.s005.tif]

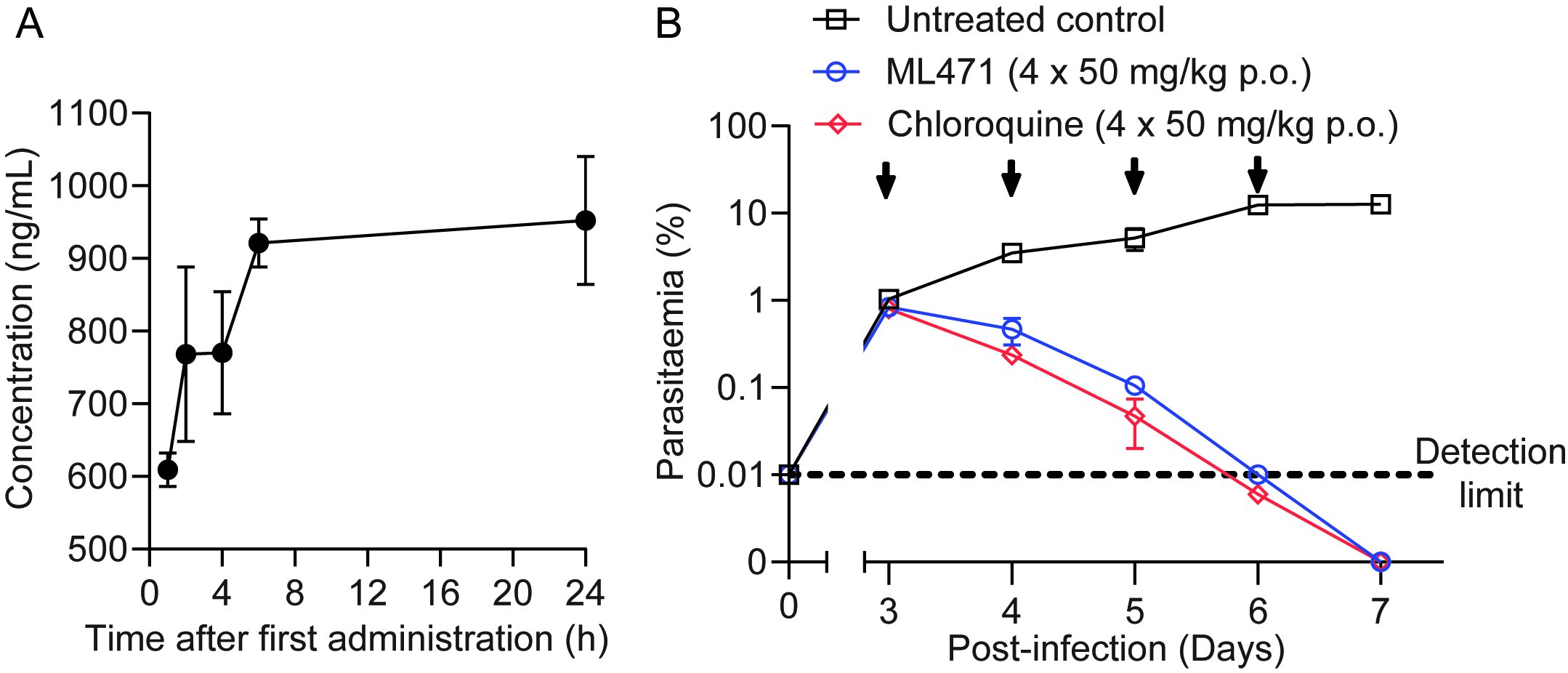

Supplement: S6 Fig — (A) Pharmacokinetics profile (in blood) for SCID mice engrafted with human RBCs infected with P. falciparum, over the first day following treatment with ML471 at 50 mg/kg p.o. See S8 Table for pharmacokinetics values. (B) Therapeutic efficacy of ML471 in the SCID mouse P. falciparum model, dosed with ML471 for 4 days at 50 mg/kg p.o. per day (arrows), initiated on Day 3 post-infection. The chloroquine data are from [16]. (TIF) [file ppat.1012429.s006.tif]

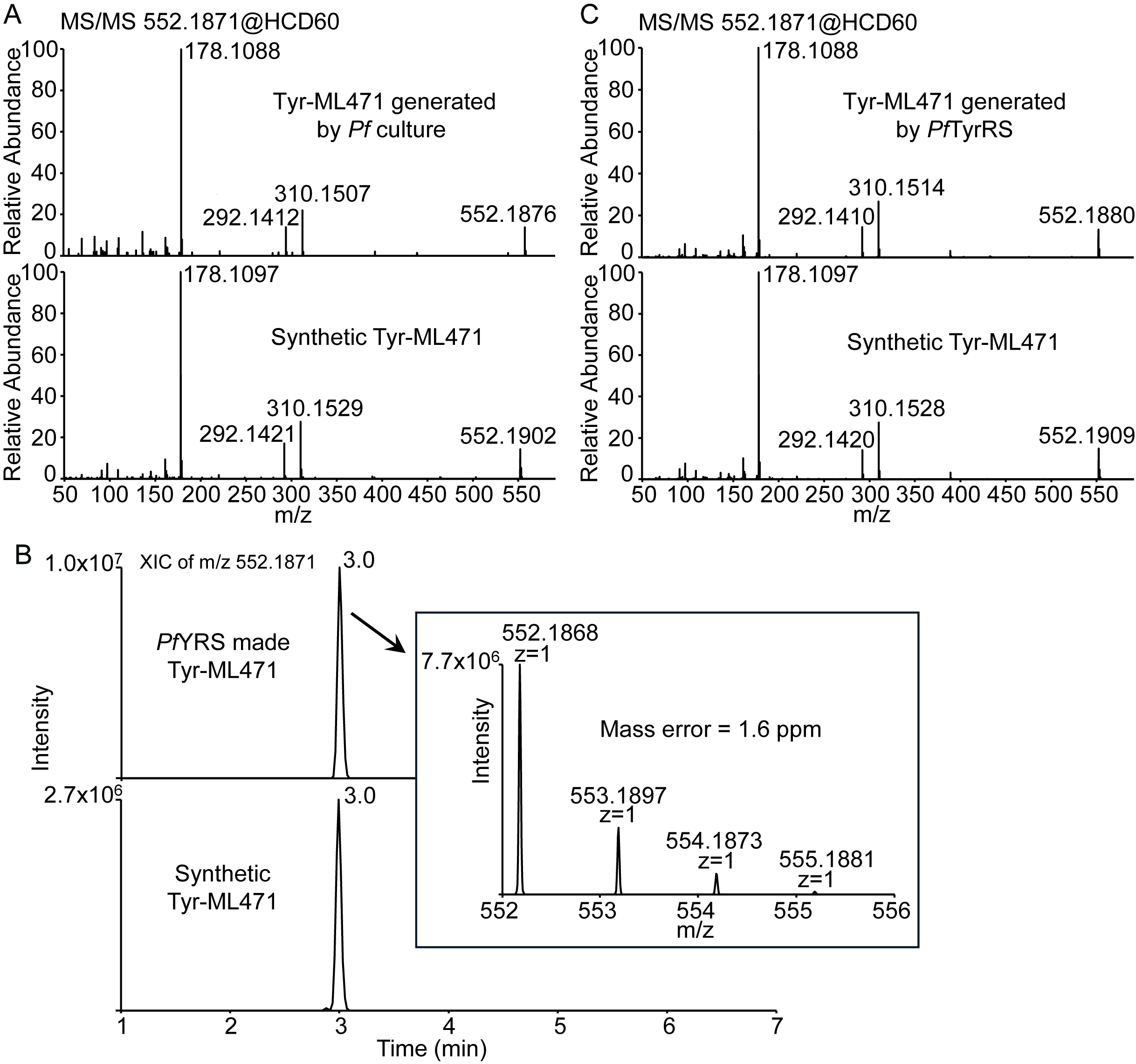

Supplement: S7 Fig — (A) MS/MS analysis of the Tyr-ML471 adduct made by P. falciparum following treatment with ML471 (1 μM) for 2 h (upper panel); and the synthetic conjugate at 0.2 μM (lower panel). (B,C) PfTyrRS (1 μM) was incubated with ML471 (10 μM), ATP (10 μM), tyrosine (20 μM) and 4 μM PftRNATyr for 1 h at 37°C. Following protein denaturation and precipitation, the supernatant was subjected to LCMS analysis. (B) The extracted ion chromatograms of Tyr-ML471 adduct made by PfTyrRS (upper panel); and the synthetic conjugate at 1 μM (lower panel). The inset shows the MS analysis of the enzyme-generated Tyr-ML471. (C) MS/MS analysis of the enzyme-generated Tyr-ML471 (upper panel) and the synthetic conjugate at 1 μM (lower panel). (TIF) [file ppat.1012429.s007.tif]

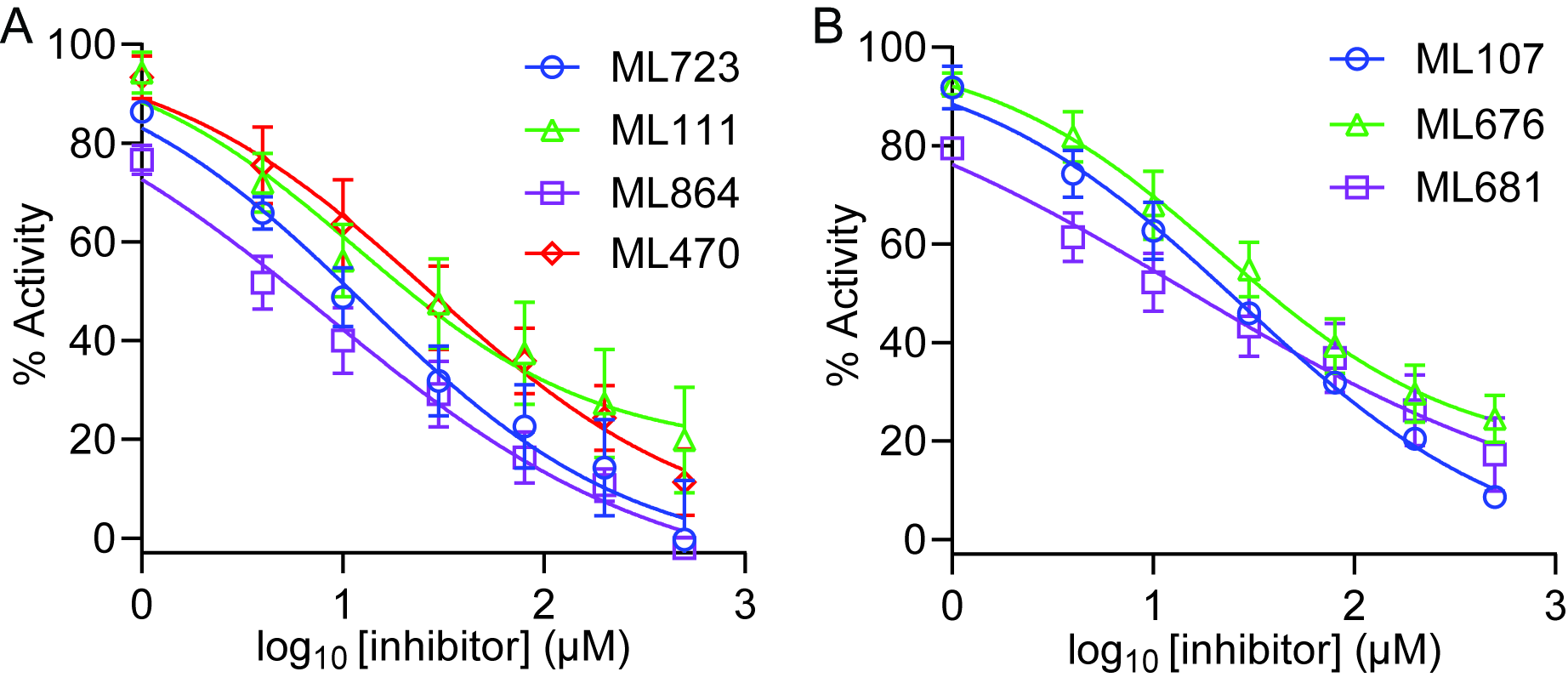

Supplement: S8 Fig — Effects of increasing concentrations of ML723, ML111, ML864, ML470 (A) and ML107, ML676, ML681 (B) on ATP consumption by PfTyrRS. The reaction conditions are: PfTyrRS (25 nM), ATP (10 μM), tyrosine (200 μM), cognate tRNATyr (4.8 μM) and pyrophosphatase (1 unit/mL). Incubations were at 37°C for 1 h. Data are mean ± SEM from three independent experiments. (TIF) [file ppat.1012429.s008.tif]

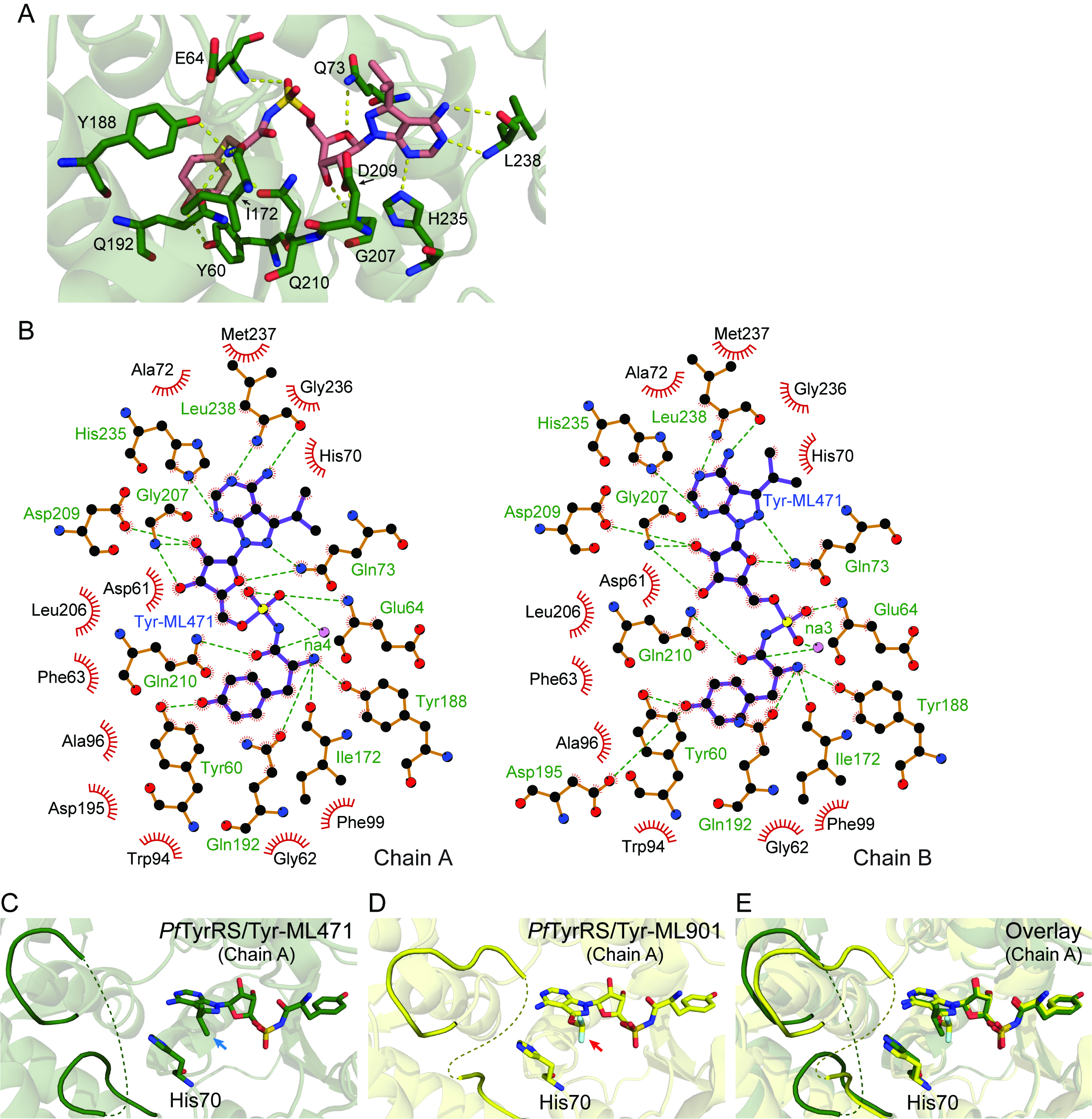

Supplement: S9 Fig — (A) Inhibitor/active site interactions for the A-chain of PfTyrRS with bound Tyr-ML471. (B) LigPlots of interacting residues for the A- and B-chains of PfTyrRS with bound Tyr-ML471. (C) A-chain of Tyr-ML471-bound PfTyrRS showing the poses adopted by the ML471 isopropyl (aqua arrow) and His70, which are incompatible with a structured KMSKS loop. (D) A-chain of Tyr-ML901-bound PfTyrRS (7ROS) illustrating the ML901 difluoromethoxy group (red arrow) and the His70 conformation. The KMSKS loop is not resolved. (E) Overlay of the A-chains of Tyr-ML471- and Tyr-ML901-bound PfTyrRS. (TIF) [file ppat.1012429.s009.tif]
