# Supplementary material for: A potent and selective reaction hijacking inhibitor of Plasmodium falciparum tyrosine tRNA synthetase exhibits single dose oral efficacy in vivo
Source: PLoS Pathog. 2024 Dec 9;20(12):e1012429. doi: 10.1371/journal.ppat.1012429 (PMC11671014; doi:10.1371/journal.ppat.1012429)
Supplement: S1 Table — (PDF) [file ppat.1012429.s010.pdf]

S1 Table. The median lethal dose (LD_{50}) values of different asexual blood stages after exposure of ML901.

n = Number of biological repeats. Data values represent mean ± SEM or range.

Drug pulse time (h)	LD_{50} (nM)	
	**Troph**	**Schizont**
**3**	1700 ± 460 (n = 3)	88 ± 1 (n = 2)
**6**	680 ± 48 (n = 3)	36 ± 6 (n = 2)
**9**	290 ± 150 (n = 3)	21 ± 12 (n = 2)
**24**	4.0 ± 0.0 (n = 3)	N/D
**48 (trophozoite), 30 (schizont), continuous**	3.9 ± 0.8 (n = 3)	8.5 ± 0.7 (n = 2)
