# Supplementary material for: A potent and selective reaction hijacking inhibitor of Plasmodium falciparum tyrosine tRNA synthetase exhibits single dose oral efficacy in vivo
Source: PLoS Pathog. 2024 Dec 9;20(12):e1012429. doi: 10.1371/journal.ppat.1012429 (PMC11671014; doi:10.1371/journal.ppat.1012429)
Supplement: S2 Table — (PDF) [file ppat.1012429.s011.pdf]

S2 Table. ***P. vivax*** **and** ***P. falciparum*** **ex vivo drug susceptibility.**

Median EC_{50} (nM) values, the range of values, in brackets, and the numbers of isolates (n) are reported.

**Compound**	**Brazilian field isolates**	
	P. falciparum	***P. vivax***
	**Median EC_{50} (nM) (Range) [Number of isolates]**	
**ML471**	4.2 (2.5-4.9) (n = 7)	8.0 (1.2-62) (n = 10)
**Artesunate**	2.2 (0.8-3.1) (n = 7)	1.7 (0.8-3.5) (n = 10)
**Chloroquine**	286 (172-520) (n = 7)	143 (104-560) (n = 10)
