# Supplementary material for: A potent and selective reaction hijacking inhibitor of Plasmodium falciparum tyrosine tRNA synthetase exhibits single dose oral efficacy in vivo
Source: PLoS Pathog. 2024 Dec 9;20(12):e1012429. doi: 10.1371/journal.ppat.1012429 (PMC11671014; doi:10.1371/journal.ppat.1012429)
Supplement: S3 Table — (PDF) [file ppat.1012429.s012.pdf]

S3 Table. **Activity against immature (>90% stage II/III) and mature (>95% stage V) stage gametocytes (*Pf*3D7-pfs16-CBG99) for ML901, ML471 and antimalarial controls.**
MB = Methylene blue. n = Number of biological repeats. Data values represent mean ± SEM.

**Compound**	*Early stage gametocytes* IC_{50} (nM)	*Mature stage gametocytes* IC_{50} (nM)
**ML901**	310 ± 60 (n = 3)	1660 ± 140 (n = 3)
**ML471**	112 ± 8 (n = 3)	392 ± 14 (n = 3)
**MMV390048**	138 ± 8 (n =3)	150 ± 20 (n = 3)
**Methylene blue**	62 ± 11 (n = 3)	353 ± 63 (n = 3)
