# Supplementary material for: A potent and selective reaction hijacking inhibitor of Plasmodium falciparum tyrosine tRNA synthetase exhibits single dose oral efficacy in vivo
Source: PLoS Pathog. 2024 Dec 9;20(12):e1012429. doi: 10.1371/journal.ppat.1012429 (PMC11671014; doi:10.1371/journal.ppat.1012429)
Supplement: S4 Table — (PDF) [file ppat.1012429.s013.pdf]

S4 Table. **Activity against liver stage *P. falciparum* schizonts and against primary hepatocytes.** Data values represent mean ± SD. n = number of independent experiments.

**Compound**	*P. falciparum liver stage schizont (NF175)* IC_{50} (nM)	*Primary hepatocytes* IC_{50} (nM)	*P. falciparum liver stage schizont (NF135)* IC_{50} (nM)	*Primary hepatocytes* IC_{50} (nM)
**ML901**	13 ± 5 (n = 3)*	>10,000 (n = 3)	2.9 ± 0.9 (n = 3)	> 100 (n = 3)
**ML471**	2.8 ± 1.5 (n = 3)	>10,000 (n = 3)	5.5 ± 2.2 (n = 3)	> 100 (n = 3)
**Atovaquone**	7.0 ± 3.9 (n = 3)	>1,000 (n = 3)	N.A.	N.A.
**MMV390048**	N.A.	N.A.	23.3 ± 13.3 (n = 3)	>100 (n = 3)
* Data from [1]				

Reference

1. Xie SC, Metcalfe RD, Dunn E, Morton CJ, Huang SC, Puhalovich T, et al. Reaction hijacking of tyrosine tRNA synthetase as a new whole-of-life-cycle antimalarial strategy. Science. 2022;376(6597):1074-9.
