# Supplementary material for: A potent and selective reaction hijacking inhibitor of Plasmodium falciparum tyrosine tRNA synthetase exhibits single dose oral efficacy in vivo
Source: PLoS Pathog. 2024 Dec 9;20(12):e1012429. doi: 10.1371/journal.ppat.1012429 (PMC11671014; doi:10.1371/journal.ppat.1012429)
Supplement: S5 Table — (PDF) [file ppat.1012429.s014.pdf]

S5 Table. Activity against transmissible gametes.
n = Number of biological repeats. Data values represent mean ± SEM.

**Compound**	Dual gamete formation assay (male) $IC_{50(48h)}$ (nM)	Dual gamete formation assay (female) $IC_{50(48h)}$ (nM)
**ML901**	130 ± 10 (n = 9)*	4,700 ± 300 (n = 9)*
**ML471**	49 ± 3 (n = 4)	260 ± 10 (n = 4)
**Cabamiquine (DDD498)**	2.3 ± 0.4 (n = 5)	2.2 ± 0.1 (n = 5)
* Data from [1]. Data for the DDD498 control is very similar to a previous report [2], validating the assay.		

References

1. Xie SC, Metcalfe RD, Dunn E, Morton CJ, Huang SC, Puhalovich T, et al. Reaction hijacking of tyrosine tRNA synthetase as a new whole-of-life-cycle antimalarial strategy. Science. 2022;376(6597):1074-9.
2. Baragaña B, Hallyburton I, Lee MCS, Norcross NR, Grimaldi R, Otto TD, et al. A novel multiple-stage antimalarial agent that inhibits protein synthesis. Nature. 2015;522(7556):315-20.
