# Supplementary material for: A potent and selective reaction hijacking inhibitor of Plasmodium falciparum tyrosine tRNA synthetase exhibits single dose oral efficacy in vivo
Source: PLoS Pathog. 2024 Dec 9;20(12):e1012429. doi: 10.1371/journal.ppat.1012429 (PMC11671014; doi:10.1371/journal.ppat.1012429)
Supplement: S6 Table — (PDF) [file ppat.1012429.s015.pdf]

S6 Table. Parasite Reduction Ratio.

Compound	Dose	Lag phase (h)	Slope	R	Log PRR	PCT99.9% (h)
**ML471**	10 x IC_{50}	0	-0.09	0.96	4.1	32
**Controls**						
**Pyrimethamine**	10 x IC_{50}	24	-0.078	0.98	3.7	56
**Chloroquine***	10 x IC_{50}	0	-0.094	0.99	4.5	33.8
**Atovaquone***	10 x IC_{50}	48	-0.060	0.99	2.9	90
**Artemisinin***	10 x IC_{50}	0	nd	nd	>4.8	<24
*Data from [1].						

Reference

1. Sanz LM, Crespo B, De-Cozar C, Ding XC, Llergo JL, Burrows JN, et al. *P. falciparum* in vitro killing rates allow to discriminate between different antimalarial mode-of-action. PloS one. 2012;7(2):e30949.
