# Supplementary material for: A potent and selective reaction hijacking inhibitor of Plasmodium falciparum tyrosine tRNA synthetase exhibits single dose oral efficacy in vivo
Source: PLoS Pathog. 2024 Dec 9;20(12):e1012429. doi: 10.1371/journal.ppat.1012429 (PMC11671014; doi:10.1371/journal.ppat.1012429)
Supplement: S7 Table — (PDF) [file ppat.1012429.s016.pdf]

**S7 Table. Pharmacological and ADME characterization of ML471.**

For PK studies, rats (n = 2) were dosed with ML471 at 1 mg/kg i.v. or 10, 20 or 25 mg/kg p.o. and plasma and blood samples were collected for analysis. For blood to plasma ratio, rats (n = 2) were dosed with ML471 at 1 mg/kg and plasma and blood samples were collected for analysis.

| **PK analysis** | | | | | | | | | | |
|---|---|---|---|---|---|---|---|---|---|---|
| **Route** | **Dose** | **Matrix** | **AUC (INF)** | **$T_{max}$** | **$C_{max}$** | **$T_{1/2z}$** | **CL** | **Vss** | **Vz** | **%F** |
| | **mg/kg** | | **(h*mM)** | **(h)** | **(nM)** | **(h)** | **(L/h/kg)** | **(L/kg)** | **(L/kg)** | |
| IV | 1 | Blood | 21.00 | 0.17 | 1180 | 36.6 | 0.138 | 6.25 | 6.54 | |
| IV | 1 | Plasma | 0.70 | 0.08 | 1540 | 2.22 | 3.84 | 2.79 | 12.3 | |
| PO | 1 | Blood | 1.95 | 16.0 | 39.3 | 23.2 | | | | 9.29 |
| PO | 1 | Plasma | 0.194 | 0.5 | 19.1 | 8.78 | | | | 27.7 |
| PO | 10 | Blood | 13.15 | 6.0 | 261 | 29.5 | | | | 9.56 |
| PO | 10 | Plasma | 1.74 | 0.38 | 196 | 9.15 | | | | 25.2 |
| PO | 25 | Blood | 29.88 | 8.0 | 669 | 30.5 | | | | 8.72 |
| PO | 25 | Plasma | 2.84 | 0.75 | 393 | 3.57 | | | | 16.4 |

| **Blood to plasma ratio** | | | | | | | |
|---|---|---|---|---|---|---|---|
| Time (h) | 0.083 | 0.25 | 0.5 | 1 | 2 | 4 | 8 |
| Mean blood to plasma ratio | 0.8 | 1.5 | 2.1 | 5.5 | 17.8 | 48.9 | 113.9 |

| **ADME data** | | | |
|---|---|---|---|
| Compound | AlogP/ TSPA | Solubility (μM) | Caco2 $P_{app}$ ($x10^{-6}$ cm/s) (A to B; B to A) |
| ML471 | -1.18/ 189 | >100 | 7.6, 0.17, 1.3. |
