# Supplementary material for: A potent and selective reaction hijacking inhibitor of Plasmodium falciparum tyrosine tRNA synthetase exhibits single dose oral efficacy in vivo
Source: PLoS Pathog. 2024 Dec 9;20(12):e1012429. doi: 10.1371/journal.ppat.1012429 (PMC11671014; doi:10.1371/journal.ppat.1012429)
Supplement: S8 Table — (PDF) [file ppat.1012429.s017.pdf]

S8 Table. Pharmacological parameters for ML471 in the SCID mouse model.
Mice were dosed orally with four daily doses of 50 mg/kg. Samples were collected from day 3, at 1, 2, 4, 6 and 24 h after the first administration. In a separate experiment, mice were dosed orally with one dose of 100 or 200 mg/kg on day 3 after infection. Samples were collected from day 3, at 1, 2, 4, 6, 24, 48, 72, 96 and 120 h after the first administration.

**SCID mouse study 4 days x 50 mg/kg p.o.**						
**Mouse**	Dose	**T_{max} [h]**	**C_{max} [ng/mL]**	**C_{max} /dose [(ng/mL)/(mg/kg)]**	**AUC0-24h [h*ng/mL]**	**AUC0-24h/dose [(h*ng/mL)/(mg/kg)]**
**M1**	50	6	888	17.8	19,586	392
**M2**	50	24	1,040	20.8	22,572	451
**N**		2	2	2	2	2
**Mean**		15	964	19.3	21,100	422
**SD**		12.7	107	2.2	2,100	42
**SCID mouse study 1 x 100 or 200 mg/kg p.o.**						
**Mouse**	Dose	**T_{max} [h]**	**C_{max} [ng/mL]**	**C_{max} /dose [(ng/mL)/(mg/kg)]**	**AUC0-120h [h*ng/mL]**	**AUC0-120h/dose [(h*ng/mL)/(mg/kg)]**
**M1**	100	4	3,100	31	239,000	2,390
**M2**	100	6	3,600	36	260,000	2,600
**N**		2	2	2	2	2
**Mean**		5	3,350	33.5	250,000	2,500
**SD**		1.4	354	3.54	14,500	145
**M3**	200	4	3,340	16.7	171,000	855
**M4**	200	4	4,180	20.9	259,000	1,290
**N**		2	2	2	2	2
**Mean**		4	3,760	18.8	215,000	1,070
**SD**		0	594	2.97	62,000	310
