# Supplementary material for: A potent and selective reaction hijacking inhibitor of Plasmodium falciparum tyrosine tRNA synthetase exhibits single dose oral efficacy in vivo
Source: PLoS Pathog. 2024 Dec 9;20(12):e1012429. doi: 10.1371/journal.ppat.1012429 (PMC11671014; doi:10.1371/journal.ppat.1012429)
Supplement: S9 Table — (PDF) [file ppat.1012429.s018.pdf]

S9 Table. Summary of resistance selection.

For ML901, Dd2-B2 parasites were subjected to pressure at 3 x IC_{50}. Mean IC_{50} = 2.6 ± 0.05 nM. For ML471, Dd2-B2 parasites were subjected to a single-step selection at 10 x IC_{50}. Mean IC_{50} and IC_{90} values were 1.45 and 1.99 nM. DSM265, a *P. falciparum* dihydroorotate dehydrogenase inhibitor, was run as a control under the same pressure conditions. The Minimum Inoculum for Resistance (MIR) value was determined as 2.1 x10^5. For comparison, compounds exhibiting favourable MIR values include MMV1091186, a *P. falciparum* isoleucyl tRNA synthetase inhibitor, with an MIR value of >10^8 [1], OSM-S-106, a *P. falciparum* asparagine tRNA synthetase inhibitor, with an MIR value of >2.4 x 10^8 [2] and MP1-12, a *P. falciparum* proteasome inhibitor, with an MIR value of >6 x 10^7 / < 2 x 10^9) [3].

**ML901**		
Number of parasites in the selection	10^7	10^8
Day of recrudescence	12 (1/3), 14 (2/3)	14 (3/3)
IC_{50} fold shift (clones)	2-3 x IC_{50}	3-6 x IC_{50}
Minimum Inoculum for Resistance (MIR)	$\leq$ 10^7	

**ML471**	
Number of parasites in the selection	2x10^5
Day of recrudescence	18 (29/96 wells)
IC_{50} shift	9-16 xIC_{50}
Minimum Inoculum for Resistance (MIR)	7.1 x10^5

References

1. Istvan ES, Guerra F, Abraham M, Huang KS, Rocamora F, Zhao H, et al. Cytoplasmic isoleucyl tRNA synthetase as an attractive multistage antimalarial drug target. Science translational medicine. 2023;15(686):eadc9249.
2. Xie SC, Wang Y, Morton CJ, Metcalfe RD, Dogovski C, Pasaje CFA, et al. Reaction hijacking inhibition of *Plasmodium falciparum* asparagine tRNA synthetase. Nat Commun. 2024;15(1):937.
3. Xie SC, Metcalfe RD, Mizutani H, Puhalovich T, Hanssen E, Morton CJ, et al. Design of proteasome inhibitors with oral efficacy in vivo against Plasmodium falciparum and selectivity over the human proteasome. Proc Natl Acad Sci U S A. 2021;118(39):e2107213118.
