# Supplementary material for: A potent and selective reaction hijacking inhibitor of Plasmodium falciparum tyrosine tRNA synthetase exhibits single dose oral efficacy in vivo
Source: PLoS Pathog. 2024 Dec 9;20(12):e1012429. doi: 10.1371/journal.ppat.1012429 (PMC11671014; doi:10.1371/journal.ppat.1012429)
Supplement: S10 Table — (PDF) [file ppat.1012429.s019.pdf]

S10 Table. Copy Number Variants (CNV) in ML901-selected sample

CNV amplification was found in sample F1 (BULK_JOS_1E7_F1), F3 (BULK_JOS_1E8_F3), F1-F6 (F1_F6_KS, a clone from the BULK 1E8 F1 selection) and FL1 (FL1_1E8) at different factors. F1 exhibits a lower increase at ~1.8 across a wider range, whereas F3, F1-F6 and FL1 show a ~2x increase only for the second half of the segment. Strikingly, three separate genome amplification boundaries were observed, with each including the tyrosine tRNA ligase (PF3D7_0807900).

**Gene ID**	**Gene Name**	**Fold gain in copy number on Chromosome 8**			
		**F1**	**F3**	**F1_F6**	**FL1**
PF3D7_0806300	ferlin-like protein, putative	1.80	1.00	1.00	1.00
PF3D7_0806400	UDP-N-acetylglucosamine transferase subunit ALG13, putative	1.80	1.00	1.00	1.00
PF3D7_0806500	DnaJ protein, putative	1.80	1.00	1.00	1.00
PF3D7_0806600	kinesin-like protein, putative	1.80	1.00	1.00	1.00
PF3D7_0806700	conserved Plasmodium membrane protein, unknown function	1.80	1.00	1.00	1.00
PF3D7_0806800	V-type proton ATPase subunit a, putative	1.80	1.00	1.00	1.00
PF3D7_0806900	conserved protein, unknown function	1.80	1.00	1.00	1.00
PF3D7_0807000	YEATS domain-containing protein, putative	1.80	1.00	1.00	1.79
PF3D7_0807100	DNA helicase PSH3	1.80	1.00	1.00	1.79
PF3D7_0807200	conserved Plasmodium membrane protein, unknown function	1.67	2.24	2.54	2.32
PF3D7_0807300	ras-related protein Rab-18	1.67	2.24	2.54	2.32
PF3D7_0807400	coenzyme Q-binding protein COQ10 homolog, mitochondrial	1.67	2.24	2.54	2.32
PF3D7_0807500	proteasome subunit alpha type-6, putative	1.67	2.24	2.54	2.32
PF3D7_0807600	conserved Plasmodium protein, unknown function	1.67	2.24	2.54	2.32
PF3D7_0807700	serine protease DegP	1.67	2.24	2.54	2.32
PF3D7_0807800	26S proteasome regulatory subunit RPN10, putative	1.67	2.24	2.54	2.32
**PF3D7_0807900**	**tyrosine tRNA ligase**	**1.67**	**2.24**	**2.54**	**2.32**
PF3D7_0808000	conserved Plasmodium protein, unknown function	1.00	2.24	2.54	2.32
PF3D7_0808100	AP-3 complex subunit delta, putative	1.00	2.24	2.54	2.32
PF3D7_0808200	plasmepsin X	1.00	2.24	2.54	2.32
PF3D7_0808300	ubiquitin regulatory protein, putative	1.00	2.24	2.54	2.32
PF3D7_0808400	coatomer subunit epsilon, putative	1.00	2.24	2.54	2.32
