# Supplementary material for: A potent and selective reaction hijacking inhibitor of Plasmodium falciparum tyrosine tRNA synthetase exhibits single dose oral efficacy in vivo
Source: PLoS Pathog. 2024 Dec 9;20(12):e1012429. doi: 10.1371/journal.ppat.1012429 (PMC11671014; doi:10.1371/journal.ppat.1012429)
Supplement: S11 Table — (PDF) [file ppat.1012429.s020.pdf]

S11 Table. Copy Number Variants (CNV) in ML471-selected sample.

CNV amplification within a 57 kb segment on chromosome 8, by ~3.8 and 4-fold in samples C2 and E2, respectively. These samples correspond to wells seeded at $2x10^5$ parasites that recrudesced following selection at 10x IC_{50}. Note that both wells had different 5' amplification breakpoints and thus were independent events that shared the same region of amplification at the 3' end. Both wells shared a ~4-fold amplification of tyrosine tRNA ligase, consistent with a slightly higher IC_{50} increase compared with ML901-selected ~2-fold amplifications described above.

Gene ID	Gene Name	Fold gain in copy number on Chromosome 8	
		C2	E2
PF3D7_0807000	YEATS domain-containing protein, putative	3.48	1.000
PF3D7_0807100	DNA helicase PSH3	3.48	1.000
PF3D7_0807200	conserved Plasmodium membrane protein, unknown function	3.80	1.000
PF3D7_0807300	ras-related protein Rab-18	3.80	1.00
PF3D7_0807400	coenzyme Q-binding protein COQ10 homolog, mitochondrial	3.80	1.00
PF3D7_0807500	proteasome subunit alpha type-6, putative	3.80	1.00
PF3D7_0807600	conserved Plasmodium protein, unknown function	3.80	1.00
PF3D7_0807700	serine protease DegP	3.80	4.04
PF3D7_0807800	26S proteasome regulatory subunit RPN10, putative	3.80	4.04
**PF3D7_0807900**	**tyrosine tRNA ligase**	**3.80**	**4.04**
PF3D7_0808000	conserved Plasmodium protein, unknown function	3.80	4.04
PF3D7_0808100	AP-3 complex subunit delta, putative	3.80	4.04
PF3D7_0808200	plasmepsin X	3.80	4.04
PF3D7_0808300	ubiquitin regulatory protein, putative	3.80	4.04
PF3D7_0808400	coatomer subunit epsilon, putative	3.80	4.04
PF3D7_0808500	Plasmodium RNA of unknown function RUF6	3.80	4.04
