# Supplementary material for: A potent and selective reaction hijacking inhibitor of Plasmodium falciparum tyrosine tRNA synthetase exhibits single dose oral efficacy in vivo
Source: PLoS Pathog. 2024 Dec 9;20(12):e1012429. doi: 10.1371/journal.ppat.1012429 (PMC11671014; doi:10.1371/journal.ppat.1012429)
Supplement: S12 Table — (PDF) [file ppat.1012429.s021.pdf]

S12 Table. T_m values of TyrRSs determined by differential scanning fluorimetry (DSF).

The T_m values for TyrRSs were measured in the apo form or after incubation with the nucleoside sulfamates in the presence of ATP, tyrosine and tRNA. AMS = Adenosine 5'-sulfamate. Data values represent mean ± SEM from three independent experiments.

*Pf*TyrRS	ML901	ML471	ML676	ML681	ML723	Apo (*Pf*TyrRS only)
T_m (°C)	65.1 ± 0.5	67.9 ± 0.4	65.5 ± 0.6	66.3 ± 0.5	66.1 ± 0.7	49.9 ± 0.6
*Pf*TyrRS	ML107	ML470	ML864	ML111	AMS	
T_m (°C)	63.5 ± 0.4	65.5 ± 0.6	66.9 ± 0.5	65.0 ± 0.5	63.5 ± 0.7	
*Hs*TyrRS	ML901	ML471	AMS	Apo (*Hs*TyrRS only)		
T_m (°C)	49.4 ± 0.1	49.0 ± 0.2	58.2 ± 0.3	49.0 ± 0.2		
