# Supplementary material for: A potent and selective reaction hijacking inhibitor of Plasmodium falciparum tyrosine tRNA synthetase exhibits single dose oral efficacy in vivo
Source: PLoS Pathog. 2024 Dec 9;20(12):e1012429. doi: 10.1371/journal.ppat.1012429 (PMC11671014; doi:10.1371/journal.ppat.1012429)
Supplement: S13 Table — (PDF) [file ppat.1012429.s022.pdf]

S13 Table. X-ray diffraction data collection and refinement statistics.

	***Pf*YRS/Tyr-ML471**
**Data collection**	
Space group	C 2
Wavelength (Å)	0.9537
Number of images	3600
Oscillation range per image (˚)	0.1
Detector	Eiger 16M
Cell dimensions	
a, b, c (Å)	138.21, 47.27, 140.512
α, β, γ (˚)	90, 94.63, 90
Resolution (Å)	46.68 - 1.80 (1.82 - 1.80)
Rsym	0.086 (0.765)
Rmeas	0.102 (0.899)
Rpim	0.054 (0.470)
CC1/2	0.998 (0.838)
I/σ(I)	9.4 (1.2)
Total observations	567540 (31557)
Unique reflections	84432 (4481)
Completeness (%)	99.9 (100.0)
Multiplicity	6.7 (7.0)
Wilson B factor (Å)	32.5
**Refinement**	
Resolution (Å)	46.68 - 1.80 (1.82 - 1.80)
Reflections used in refinement	84394 (2835)
Rfree reflections	4177 (136)
R_{work}	0.1830 (0.3172)
R_{free}	0.2198 (0.3517)
Protein molecules in asymmetric unit	2
Total nonhydrogen atoms	6159
Protein	5683
Ligand/ion	96
Solvent	380
Mean B factor ($Å^2$)	50.31

Protein	50.50
Ligand/ion	39.63
Solvent	50.14
RMS deviations	
Bond lengths (Å) (outliers > 4 σ)	0.017
Bond angles (°) (outliers > 4 σ)	1.52
Rotamer outliers (%)	1.09
Clashscore	6.33
Cβ outliers	0
Molprobity score	1.35
Ramachandran Plot	
Favoured (%)	98.55
Allowed (%)	1.45
Outliers (%)	0
