# Supplementary material for: A potent and selective reaction hijacking inhibitor of Plasmodium falciparum tyrosine tRNA synthetase exhibits single dose oral efficacy in vivo
Source: PLoS Pathog. 2024 Dec 9;20(12):e1012429. doi: 10.1371/journal.ppat.1012429 (PMC11671014; doi:10.1371/journal.ppat.1012429)
Supplement: S1 Text — (DOCX) [file ppat.1012429.s023.docx]

**S1 Text. Chemistry materials and methods.**

*Chemical abbreviations*

Boc: *tert*-Butoxycarbonyl

DIPEA: *N,N*-Diisopropylethylamine

DMA: *N,N*-Dimethylacetamide

DMAP: 4-Dimethylaminopyridine

DMF: *N,N*-Dimethylformamide

DMSO: Dimethyl sulfoxide

EDCI: 1-Ethyl-3-(3-dimethylaminopropyl) carbodiimide

HPLC: High-performance liquid chromatography

LC-MS: Liquid chromatography-mass spectrometry

NIS: *N*-Iodosuccinimide

NMR: Nuclear magnetic resonance spectroscopy

*p*-TsOH: *p*-Toluenesulfonic acid

TFA: Trifluoroacetic acid

THF: Tetrahydrofuran

TPPTS: Triphenylphosphine-3,3′,3′′-trisulfonic acid trisodium salt

Tyr: Tyrosine

*General information*

Reagents were purchased from Sigma-Aldrich, Merck, Fisher Scientific, and Combi-Blocks, and were used without further purification. Anhydrous conditions: glassware was dried at >130 °C for >12 h, assembled hot, and purged with nitrogen (N_2_) gas where suitable. Reduced pressure/*in vacuo* implies 900 to 50 mbar under rotary evaporation at 40–50 °C.

Liquid chromatography-mass spectrometry (LC-MS) data were documented on an Agilent 1260 Infinity II LC System with a Diode Array HS (G7117C) UV-Visible detector coupled to a triple quadrupole mass detector. (analytical column: Pursuit XR C18 100 Å, 3 μm, 2 mm × 50 mm; solvent gradient: 5–95% acetonitrile in 0.05% v/v aqueous trifluoroacetic acid; flow rate: 0.4 ml min^-1^; elution run time: 14 minutes). The charge of the ion specifies that the detection is positive; for instance, [M+H]^+^ denotes positive-ion detection.

Analytical thin-layer chromatography (TLC) was carried out on Merck Silica Gel 60 F_254_-precoated aluminum plates (0.2 mm) and observed using UV irradiation (254 nm and 280 nm). High-temperature reactions were carried out in DrySyn heating blocks.

Proton (^1^H) NMR spectra were recorded on a BrukerDRX400 spectrometer operating at 400 MHz for proton nuclei. Deuterated solvents (CDCl_3_, CD_3_OD and DMSO-*d_6_*) were obtained from Sigma-Aldrich. ^1^H chemical shifts are stated in parts per million (ppm). Spectroscopic chemical shifts were calibrated to residual solvent peaks (^1^H: CHCl_3_ 7.26 ppm, methanol 3.31ppm, dimethyl sulfoxide 2.50 ppm). The multiplicities are labelled as either a singlet (s), doublet (d), triplet (t), doublet of doublets (dd), or multiplet (m).

For preparative HPLC purification, samples were injected onto a Phenomenex Luna C8 column (5 μM particle size, 100 Å, 150 x 21.2 mm) and analysed on Agilent Technologies 1260 Infinity II HPLC system equipped with a photodiode array detector, and a preparative fraction collector (G1364E), (Solvent gradient: 2–35% acetonitrile in 0.05% v/v aqueous trifluoracetic acid; flow rate: 15 ml min^-1^; elution run time: 40 minutes).

**Synthesis and Characterisation of *ML471 and Tyr-ML471***


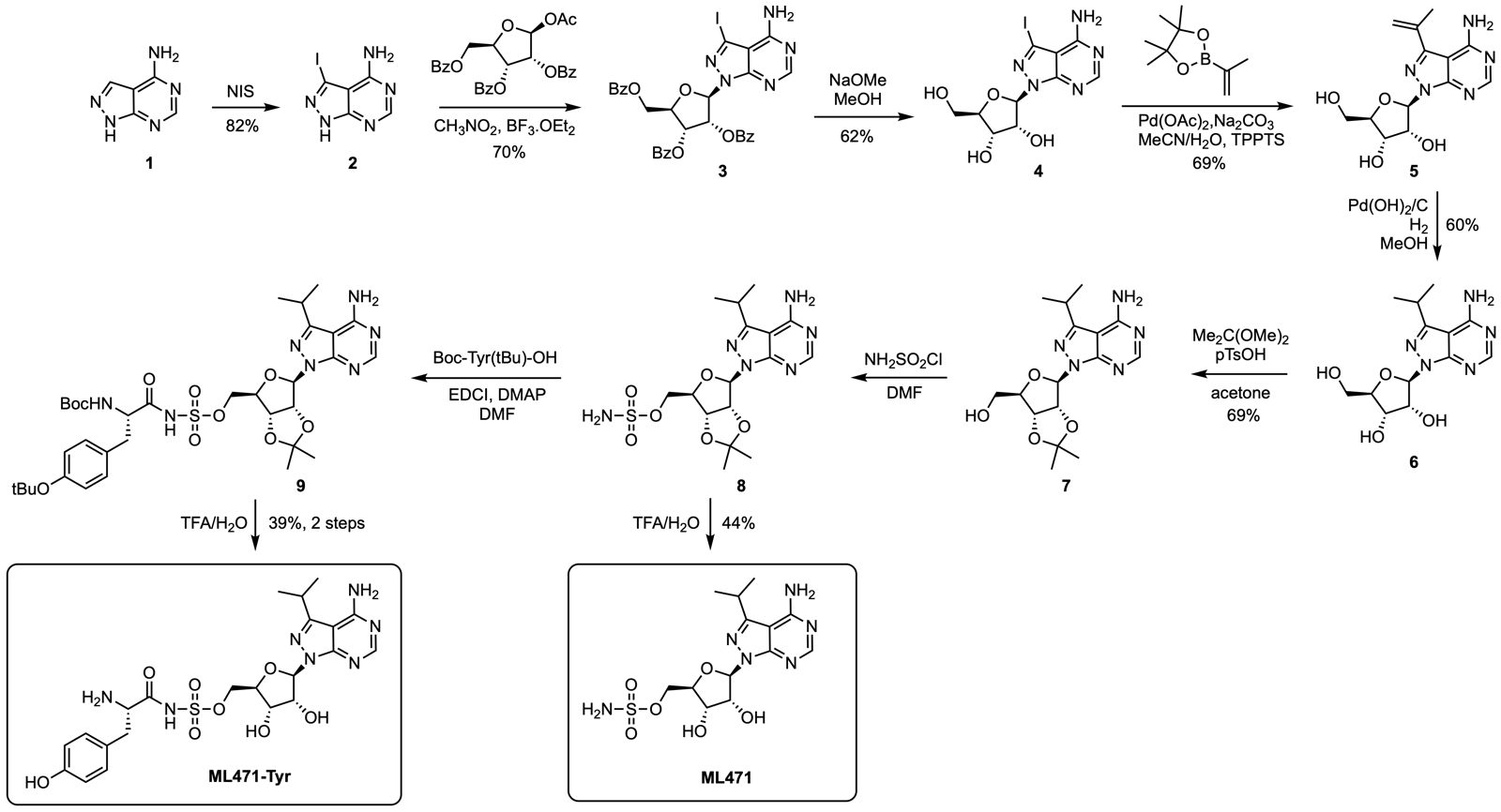


**Scheme 1. Synthetic routes to ML471 and Tyr-ML471.**

*3-iodo-1H-pyrazolo[3,4-d]pyrimidin-4-amine (****2****).*

4-Amino-1*H*-pyrazolo[3,4-*d*]pyrimidine (**1**, 5.0 g, 37 mmol) was dissolved in DMF (40 mL). *N‑*Iodosuccinimide (NIS) (9.16 g, 40.7 mmol) was added, and the mixture was heated at 80 °C overnight. The mixture was cooled to room temperature and poured into ice-cold water (350 mL). The resulting suspension was stirred for 10 min at 0 °C then filtered. The solids were collected and dried *in vacuo* to afford **2** (8.8 g, 82% yield) as a white solid. ^1^H NMR (300 MHz, DMSO-*d*_6_) δ 8.16 (1H, s), 13.80 (1H, br s) ppm. LC-MS: *m/z* 261.95 (M+H^+^). Characterization data are in accordance with reported values [1].

*(2R,3R,4R,5R)-2-(4-amino-3-iodo-1H-pyrazolo[3,4-d]pyrimidin-1-yl)-5-((benzoyloxy)methyl)tetrahydrofuran-3,4-diyl dibenzoate (****3****).*

The pyrazolopyrimidine **2** (1.0 g, 3.83 mmol) and 1-*O*-acetyl-tri-*O*-benzoyl-β-D-ribofuranose (2.32 g, 4.6 mmol) were added to dry nitromethane (10 mL). The mixture was heated at reflux, then BF_3_·OEt_2_ (0.6 mL) was added, upon which the solids started to dissolve. After heating at reflux for 90 min, the solvent was removed *in vacuo*. The resulting oil was purified by silica column chromatography: excess 1-*O*-acetyl-tri-*O*-benzoyl-β-D-ribofuranose was eluted with 100% CH_2_Cl_2_, then elution with 5% acetone in CH_2_Cl_2_ afforded the product **3** (1.9 g, 70% yield) as a pale brown oil. ^1^H NMR (300 MHz, CDCl_3_) δ 4.56–4.90 (3H, m), 6.20 (1H, t, *J* = 5.6 Hz), 6.36 (1H, dd, *J* = 5.0, 3.2 Hz), 6.76 (1H, d, *J* = 2.9 Hz), 7.35–7.62 (9H, m), 7.91–8.14 (6H, m), 8.34 (1H, s) ppm. LC-MS: *m/z* 706.07 (M+H^+^). Characterization data are in accordance with reported values [1].

*(2R,3R,4S,5R)-2-(4-amino-3-iodo-1H-pyrazolo[3,4-d]pyrimidin-1-yl)-5-(hydroxymethyl)tetrahydrofuran-3,4-diol (****4****).*

Protected nucleoside **3** (1.15 g, 1.63 mmol) was dissolved in CH_2_Cl_2_ (0.85 mL). Methanol (8.5 mL) was added, followed by a solution of NaOMe in MeOH (4.5 M, 0.39 mL). The mixture was stirred at 60 °C for 2 h. The reaction was neutralised to pH 7 by the addition of 4 N HCl, and the solvents were removed *in vacuo*. The solid residue was purified by silica column chromatography (5–15% MeOH/CH_2_Cl_2_ gradient) to afford the final product **4** (0.400 g, 62% yield). ^1^H NMR (300 MHz, DMSO-*d*_6_) δ 3.43 (1H, dd, *J* = 11.7, 5.6 Hz), 3.55 (1H, dd, *J* = 11.7, 4.4 Hz), 3.89 (1H, dd, *J* = 10.0, 4.4 Hz), 4.16 (1H, t, *J* = 4.7 Hz), 4.57 (1H, m), 6.03 (1H, d, *J* = 5.0 Hz), 8.23 (1H, s) ppm. LC-MS: *m/z* 394.00 (M+H^+^). Characterization data are in accordance with reported values [1].

*(2R,3R,4S,5R)-2-(4-amino-3-(prop-1-en-2-yl)-1H-pyrazolo[3,4-d]pyrimidin-1-yl)-5-(hydroxymethyl)tetrahydrofuran-3,4-diol (****5****).*

Compound **4** (500 mg, 1.27 mmol) was added to 2-isopropenylboronic acid pinacol ester (317 mg, 3.81 mmol), Na_2_CO_3_ (405 mg 3.81 mmol), Pd(OAc)_2_ (14.3 mg, 0.065 mmol), and TPPTS (87.4 mg, 0.15 mmol). Acetonitrile (2.5 mL) and H_2_O (5 mL) were added to the solids under nitrogen. After 5 min of stirring, the mixture was heated at reflux. Upon completion, the mixture was cooled to room temperature and neutralised to pH ∼7 with 4 M aq. HCl. The mixture was concentrated *in vacuo* and the residue was purified by silica column chromatography (2–15% MeOH/CH_2_Cl_2_ gradient) to afford **5** (270 mg, 69% yield) as an off-white solid. ^1^H NMR (300 MHz, DMSO-*d*_6_) δ 2.18 (3H, s), 3.45 (1H, m), 3.60 (1H, m), 3.92 (1H, dd, *J* = 9.7, 4.4 Hz), 4.23 (1H, dd, *J* = 9.7, 4.7 Hz), 4.59 (1H, dd, *J* = 9.4, 5.0 Hz), 4.84 (1H, t, *J* = 5.6 Hz), 5.12 (1H, d, *J* = 5.3 Hz), 5.29–5.45 (2H, m), 5.53 (1H, s), 6.13 (1H, d, *J* = 4.1 Hz), 8.23 (1H, s) ppm. LC-MS: *m/z* 308.14 (M+H^+^). Characterization data are in accordance with reported values [1].

*(2R,3R,4S,5R)-2-(4-amino-3-isopropyl-1H-pyrazolo[3,4-d]pyrimidin-1-yl)-5-(hydroxymethyl)tetrahydrofuran-3,4-diol (****6****).*

Compound **5** (500 mg, 1.62 mmol) was dissolved in MeOH (10 mL) and Pd(OH)_2_/C (50 mg) was added. The vessel was filled with H_2_ at 20 psi, and the mixture was stirred overnight. The mixture was filtered through celite, and the solvents were removed *in vacuo*. The solid residue was purified by silica column chromatography (4–20% MeOH/CH_2_Cl_2_ gradient) to afford **6** (300 mg, 60% yield) as a white solid. ^1^H NMR (300 MHz, DMSO-*d*_6_) δ 1.26 (6H, d, *J* = 6.7 Hz), 3.38–3.65 (3H, m), 3.90 (1H, dd, *J* = 9.4, 4.7 Hz), 4.25 (1H, dd, *J* = 10.3, 5.0 Hz), 4.56 (1H, dd, *J* = 10.0, 5.3 Hz), 4.85 (1H, dd, *J* = 6.7, 5.0 Hz), 5.07 (1H, d, *J* = 5.6 Hz), 5.32 (1H, d, *J* = 5.9 Hz), 6.06 (1H, d, *J* = 4.4 Hz), 8.15 (1H, s) ppm. LC-MS: *m/z* 310.14 (M+H^+^). Characterization data are in accordance with reported values [1].

*((3aR,4R,6R,6aR)-6-(4-amino-3-isopropyl-1H-pyrazolo[3,4-d]pyrimidin-1-yl)-2,2-dimethyltetrahydrofuro[3,4-d][1,3]dioxol-4-yl)methanol (****7****).*

To compound **6** (570 mg, 1.84 mmol) in acetone (25 mL) was added 2,2-dimethoxypropane (1.13 mL, 9.2 mmol) and *p*-TsOH (350 mg, 1.84 mmol) and the mixture was stirred overnight at room temperature. Chilled aq. sat. NaHCO_3_ was added and the mixture was stirred for 5 minutes, then dried *in vacuo*. The residue was dissolved by stirring in acetone for 1 h, the mixture was filtered and the filtrate evaporated *in vacuo*. The residue was purified by silica column chromatography (5% EtOH in EtOAc) to afford **7** (450 mg, 69% yield). ^1^H NMR (300 MHz, DMSO-*d*_6_) δ 1.26 (6H, d, *J* = 6.7 Hz), 1.32 (3H, s), 1.51 (3H, s), 3.39 (1H, m), 3.50–3.62 (2H, m), 4.13 (1H, m), 4.94 (1H, d, *J* = 6 Hz), 5.23 (1H, m), 6.26 (1H, d, *J* = 1.8 Hz), 8.16 (1H, s) ppm. LC-MS: *m/z* 350 (M+H^+^). Characterization data are in accordance with reported values [2].

*((3aR,4R,6R,6aR)-6-(4-amino-3-isopropyl-1H-pyrazolo[3,4-d]pyrimidin-1-yl)-2,2-dimethyltetrahydrofuro[3,4-d][1,3]dioxol-4-yl)methyl sulfamate (****8****).*

To compound **7** (425 mg, 1.21 mmol) in dry DMF (4.5 mL) was added sulfamoyl chloride (421 mg, 3.64 mmol) and the mixture was stirred at room temperature for 2 h. Several drops of methanol were added and the solvent was evaporated *in vacuo*. The compound was used in the next step without further purification. LC-MS: *m/z* 429.1 (M+H^+^).

*((2R,3S,4R,5R)-5-(4-amino-3-isopropyl-1H-pyrazolo[3,4-d]pyrimidin-1-yl)-3,4-dihydroxytetrahydrofuran-2-yl)methyl sulfamate (****ML471****)*

Compound **8** (100 mg, 0.23 mmol) was dissolved in TFA/H_2_O (4:2). The mixture stirred at room temperature overnight, and completion of the deprotection was confirmed by LC-MS. The solvents were evaporated *in vacuo* and the residue was purified by HPLC (C8 column, 2–35% acetonitrile/H_2_O) to afford the title compound **ML471** (40 mg, 44% yield) as a white solid. ^1^H NMR (400 MHz, CD_3_OD) δ 1.40 (6H, t, *J* = 7.0 Hz), 3.48 (1H, h, *J* = 6.8 Hz), 4.20–4.31 (2H, m), 4.35 (1H, dd, *J* = 10.2, 3.1 Hz), 4.63–4.68 (2H, m), 6.34 (1H, d, *J* = 1.9 Hz), 8.35 (1H, s). LC-MS: *m/z* 389.2 (M+H^+^). Characterization data are in accordance with reported values [2].

*((3aR,4R,6R,6aR)-6-(4-amino-3-isopropyl-1H-pyrazolo[3,4-d]pyrimidin-1-yl)-2,2-dimethyltetrahydrofuro[3,4-d][1,3]dioxol-4-yl)methyl ((S)-3-(4-(tert-butoxy)phenyl)-2-((tert-butoxycarbonyl)amino)propanoyl)sulfamate (****9****).*

Compound **8** (50 mg, 0.12 mmol) was added to Boc-Tyr(*t*Bu)-OH (60.7 mg, 0.18 mmol), EDCI (92.0 mg, 0.48 mmol), DMAP (16.1 mg, 0.13 mmol) in dry DMF (5 mL) and the mixture was stirred overnight at room temperature. The solvent was evaporated *in vacuo*. The residue was added to water and the organic layer was extracted using EtOAc, dried using Na_2_SO_4_, and evaporated *in vacuo* to give a crude sample of **9** (100 mg), a viscous pale brown oil, which was used in the next step without further purification. LC-MS: *m/z* 748.1 (M+H^+^).

*((2R,3S,4R,5R)-5-(4-amino-3-isopropyl-1H-pyrazolo[3,4-d]pyrimidin-1-yl)-3,4-dihydroxytetrahydrofuran-2-yl)methyl (L-tyrosyl)sulfamate (****Tyr-ML471****)*

The crude compound **9** (100 mg) was dissolved in TFA (0.8 mL) and CH_2_Cl_2_ (4 mL) and the mixture was stirred at room temperature for 2 h. LC-MS confirmed deprotection of the tBu and Boc groups (*m/z* 592 (M+H)). Water (0.4 mL) was added, and the mixture was stirred at room temperature overnight. The volatiles were removed *in vacuo* then the residue was purified by column chromatography (5–15% MeOH/CH_2_Cl_2_ gradient), followed by HPLC purification (C8 column, 2–35% acetonitrile/H_2_O) to afford the title compound **Tyr-ML471** (25 mg, 34% yield over 2 steps) as an off-white solid. ^1^H NMR (400 MHz, DMSO-*d_6_*) 1.26 (6H, d, *J* = 6.7 Hz), 2.64–2.88 (2H, m), 3.02 (1H, m), 3.98–4.12 (2H, m), 4.24 (1H, m), 4.37 (1H, t, *J* = 5.1 Hz), 4.51 (1H, m), 6.13 (1H, d, *J* = 3.0 Hz), 6.64–6.74 (2H, m), 7.01–7.05 (2H, m), 8.27 (1H, s). LC-MS: *m/z* 552.2 (M+H^+^).


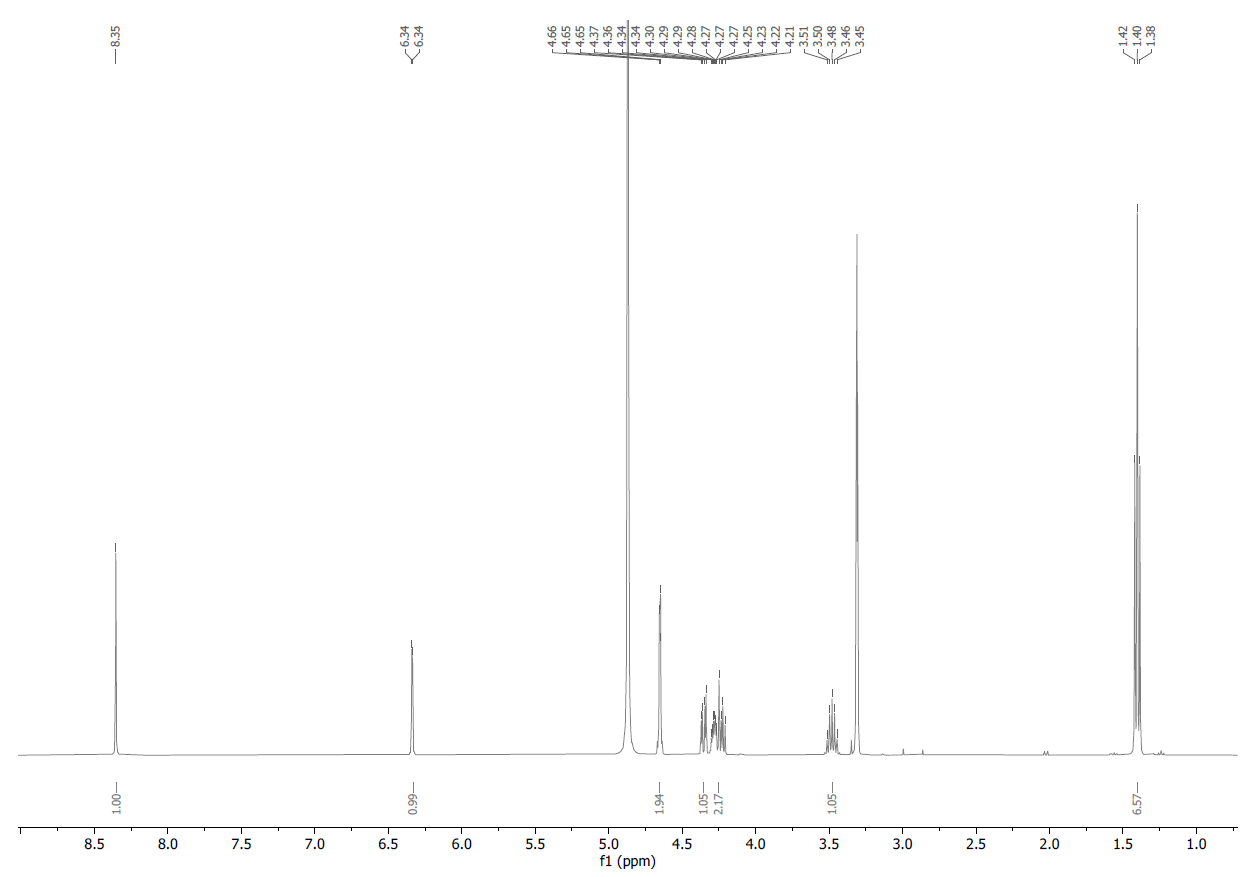

**^1^H NMR Spectrum of ML471.**


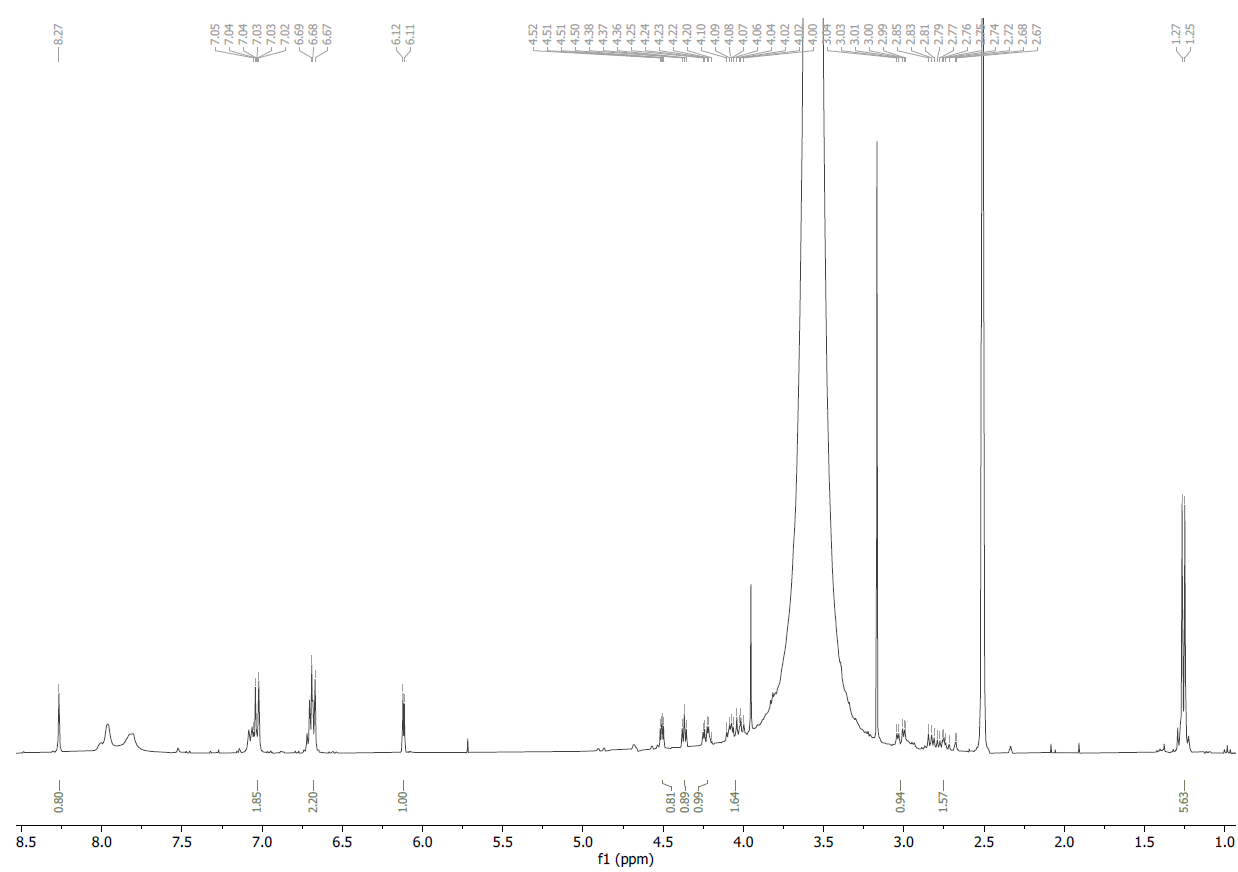

**^1^H NMR Spectrum of Tyr-ML471.**

**References**

1. Bouton J, Ferreira de Almeida Fiuza L, Cardoso Santos C, Mazzarella MA, Soeiro MdNC, Maes L, et al. Revisiting pyrazolo[3,4-d]pyrimidine nucleosides as anti-*Trypanosoma cruz*i and antileishmanial agents. Journal of medicinal chemistry. 2021;64(7):4206-38.

2. Adhikari S, Calderwood EF, England DB, Gould AE, Harrison SJ, Huang S-C, et al. Atg7 inhibitors and the uses thereof. World Intellectual Property Organization. 2017;WO/2018/089786 PCT/US2017/061094.
