# Supplementary material for: A potent and selective reaction hijacking inhibitor of Plasmodium falciparum tyrosine tRNA synthetase exhibits single dose oral efficacy in vivo
Source: PLoS Pathog. 2024 Dec 9;20(12):e1012429. doi: 10.1371/journal.ppat.1012429 (PMC11671014; doi:10.1371/journal.ppat.1012429)
Supplement: Attachment — Submitted filename: Response to reviewers comments_Oct 16.docx [file ppat.1012429.s024.docx]

PLOS Pathogens

Audrey Odom John, MD, PhD Academic Editor

Tracey Lamb, Section Editor

Michael Malim, Editor-in-Chief

Dear Colleagues

Thank you for your email of September 20, regarding our submission PPATHOGENS-D-24-01523

“A potent and selective reaction hijacking inhibitor of Plasmodium falciparum tyrosine tRNA synthetase exhibits single dose oral efficacy in vivo".

Thank you for indicating that PLOS Pathogens is likely to accept the manuscript for publication, if we can modify the manuscript according to the review recommendations.

We have addressed the review recommendations as detailed below; and provided a description of the changes we have made in the manuscript.

We have uploaded two versions of the revised manuscript: one with tracked changes denoting where the text has been changed; and a clean version.

We hope that the manuscript is now ready for publication in PLoS Pathogens.

Kind regards

Steve Langston, Mike Griffin and Leann Tilley

-

***Part I - Summary*** *Please use this section to discuss strengths/weaknesses of study, novelty/significance, general execution and scholarship.*

*Reviewer #1: In the manuscript titled “A potent and selective reaction hijacking inhibitor of Plasmodium falciparum tyrosine tRNA synthetase exhibits single dose oral efficacy in vivo,” Xie et al explore ML901 derivatives to identify a candidate with improved selectivity for future antimalarial development. This led to the discovery of ML471, which exhibits activity against the P. falciparum asexual blood stage, sexual stage and liver stage. Activity against clinical isolates (falciparum and vivax) as well as drug resistant P. falciparum was confirmed. The possibility that ML901 exhibits cytotoxicity due to human E1 targeting was explored and ML471 was found to have decreased targeting of human UAE. ML471 was tested in an in vivo mouse malaria model where it had single dose oral efficacy against P. falciparum and rapid killing, meeting current MMV recommendations for new candidate selection criteria. Though it is acknowledged that it does not meet goals for reduced propensity of resistance. Lastly, structural studies were completed that provide insights into the mechanism of selectivity. The experiments presented are robust, and the authors nicely frame their findings in the context of previously published work and the current state of targeting tRNA synthetases. This impactful and timely manuscript will be of broad interest to the pathogen and drug development communities.*

We thank the Reviewer for their kind comments.

*Reviewer #2: The manuscript is very well written and serves to be a very good example of the strength of collaborative work. The authors have indeed done a commendable amount of work and have very clearly justified every aspect of the compound testing that they performed. All of their claims are very well justified with the experimental results they have. The significance of this work can be observed by virtue of the establishment of a whole “pipeline” to effectively scrutinize multiple aspects of a compound in drugging the malarial parasite. This work, with its immense width-and-depth analyses positions itself to form a basis of future works utilizing the concept of reaction-hijacking prodrugs in more use-cases. The novelty of chemical classes being discovered and synthesized to have desirable bioactive profiles promises to continue to be an evergreen reiterative process to push the limits of molecular medicine.*

We thank the Reviewer for their kind comments.

**Part II – Major Issues: Key Experiments Required for Acceptance**
***Reviewer #1***

*i) Figure 4A, could a control (no drug) trace be added to demonstrate no peak is observed in parasites without compound addition.*

We have now included a control (no drug) trace as part of Fig 4A.

*ii) The magnitude of a Tm shift (driven by thermodynamics) is not proportional with ligand binding affinity (pg 10, line 248). Perhaps reword this to discuss a more stable conformational change induced by the small molecule. Alternatively, ITC or MST could be used to measure the Kd.*

Line 248 (now Line 267) has been changed to read: “ The increase in thermal stability is even greater than that induced by the Tyr-ML901 adduct (Table 3, S12), which is consistent with the enhanced antimalarial activity of ML471 compared with ML901.”

*It is also recommended to remove this suggestion (that enhanced thermal stability indicated tighter binding) in line 337.*

Line 337 (now Line 361) has been changed to read: “The improved potency derives from improved efficiency of reaction hijacking inhibition of *Pf*TyrRS and is consistent with enhanced thermal stability induced by the Tyr-ML471 adduct”.

Reviewer #2: No major issues detected in the manuscript.

***Part III – Minor Issues: Editorial and Data Presentation Modifications*** *Reviewer #1:*

*i) Is there a structural rationale for the 8 analogs tested?*

Initial studies indicated that different substitutions at the 7-position of the pyrazolopyrimidine ring system were tolerated but were associated with different levels of potency and selectivity. We therefore explored a broad selection of analogues with different 7-position substituents that were available in the Takeda nucleoside sulfamate library.

*ii) Can the authors speculate on the loss of efficacy observed between the sexual and asexual stage?*

Similar differential potency against asexual blood stage and gametocytes has been observed previously for an inhibitor of *P. falciparum* cysteine tRNA synthetase, MMV1091186 (Istvan et al., 2023) and an inhibitor of phenylalanyl-tRNA synthetase BRD3444 (Kato et al., 2016). This is likely due to the fact that gametocytes develop more slowly and do not undergo cell division; and are therefore less susceptible to inhibition of protein translation. In this context, it is interesting to note that, as exemplified in Fig S1, the schizont stage of the asexual blood stage is more sensitive than the trophozoite stage, which may reflect the need for rapid synthesis of daughter merozoite proteins.

In an effort to address the reviewer’s question we have now included an additional sentence (Line 180) that reads: “The lower potency against gametocytes compared with asexual blood stage may reflect the fact that gametocytes develop more slowly and are therefore less susceptible to inhibition of protein translation.”

*iii) Also, has the activity of ML471 or ML901 on parasite invasion been investigated?*

The effects of ML471 or ML901 on parasite invasion was not specifically investigated in this study; but the potent killing of schizont stage parasites suggests that the viability of nascent merozoites is strongly inhibited.

*iv) pg 7, line 107 could the notation be added to indicate it was at 72 hr for the IC50.*

Done.

*v) On pg 9, line 210 it is stated that dosing led to “no evidence of toxicity.” Could the assessment for toxicity be clarified?*

Line 541. A sentence has been added to clarify possible symptoms of toxicity. “Animal were monitored for symptoms of acute toxicity, including decreased activity, sunken flanks, ataxia and blood in faeces.”

*vi) Could Table S9 include previously published metrics for compounds that have favorable Minimum Inoculum for Resistance values for comparison?*

We have now included reference to some literature data for compounds with different MIR values that were assessed under similar conditions to the ones used in this work.

The following information is provided in the figure legend to Table S9. “For comparison, compounds exhibiting favourable Minimum Inoculum for Resistance (MIR) values include MMV1091186, a *P. falciparum* isoleucyl tRNA synthetase inhibitor, with an MIR value of >10^8^ (Istvan et al., 2023), OSM-S-106, a *P. falciparum* asparagine tRNA synthetase inhibitor, with an MIR value of >2.4 x 10^8^ (Xie et al., 2024) and MP1-12, a *P. falciparum* proteasome inhibitor, with an MIR value of >6 x 10^8^ / < 2 x 10^9^) (Xie et al., 2021).

*vii) These compounds bind/modify the protein in the nM range but inhibit ATP consumption in the uM range. Could a rational for this be included?*

We have now included an explanation (Line 282). “The concentration of ML471 needed to induce 50% inhibition of ATP consumption (1.4 μM) is much higher than the amount needed to kill parasites in a 72-h exposure assay (1.5 nM). In the biochemical assay, the recombinant enzyme generates a charged tRNA product, which is then attacked by ML471 to generate the inhibitory Tyr-ML471 adduct. By contrast, in cells, tRNAs are generally fully loaded (Evans et al., 2017), which is expected to promote adduct formation. In addition, binding of sulfamate-containing compounds to carbonic anhydrase (Boddy et al., 1989; Supuran, 2010) will facilitate accumulation of ML471 into infected red blood cells.”

*viii) Conventionally prodrugs (or proinhibitors) require modification by cytochrome P450s, or other enzymes, to yield active molecules. Here, it seems pro-inhibitor is indicating the molecule pre modification of the target? To the best of my knowledge, this terminology has not been used for covalent kinase inhibitors.*

We accept the Reviewer’s point. We have removed reference to pro-inhibitors and instead used the term reaction hijacking inhibitor or simply nucleoside sulfamate.

Reviewer #2:

i) Line 218 – For the in vitro resistance evolution experiment, what was the rationale for the authors to use two differential methods of inducing resistance?

These resistance selections were performed at different time points, over one year apart, and used standardised protocols in use at the time of the experiment. A statement has been added to the Methods: “The different pressure regimens reflect the standardised protocol used in the Fidock lab at the times of analysis.”

ii) Line 587 – Please mention the ratio of “Enzyme: Compound” for both the enzymes tested and the associated rationale of performing the assay at different ratios for the Pf and Hs enzymes. Currently only the PfTyrRS parameters are mentioned.

We apologise for this oversight. An additional sentence has now been added to the Methods section (Line 618). “Alternatively, *Hs*TyrRS (2.3 μM) was incubated with 200 μM nucleoside sulfamate (molar ratio 1: 87) with 10 μM ATP, 20 μM L-tyrosine, 8 mg/mL yeast tRNA, at 37°C for 4 h. A longer incubation and higher nucleoside sulfamate concentration was used for *Hs*TyrRS, given its weaker susceptibility to hijacking.”

*iii) Line 761 – Please provide a full legend for the Table contents as to what is each column value depicting.*

The Table legend has been expanded and now reads:

**Table 1. Activities of pyrazolopyrimidine sulfamates as inhibitors of parasite growth.** *P. falciparum* (3D7, ring stage) cultures were exposed to different nucleoside sulfamates for 72 h and the 50% Inhibitory Concentration (IC_50_) or growth inhibition assessed using the lactate dehydrogenase (*Pf*LDH) growth inhibition assay. Alternatively, synchronized Cam3.II^rev^ parasite cultures were subjected to 6-h pulses of nucleoside sulfamates, at the trophozoite (25-30 h.p.i.) stage. 50% growth inhibition (IC_50_) was determined in the cycle following treatment, using a SYBR Green I assay. HepG2 cell cultures were exposed to nucleoside sulfamates for 72 h and growth inhibition (IC_50_) assessed using CellTiter-Glo reagent. Data represent the mean of three independent experiments and error bars correspond to SEM. AMS = Adenosine 5'-sulfamate. n = Number of biological repeats. Data values represent mean ± SEM. Medicines for Malaria Venture (MMV) designations for the compound names are in brackets.

*iv) Line 766 – Please provide a full legend for the Table contents as to what is each column value depicting.*

The Table legend has been expanded and now reads:

**Table 2. Inhibitory activity of selected pyrazolopyrimidine sulfamates in E1 enzymes assays.** Homogeneous Time-Resolved Fluorescence (HTRF) enzyme assays were employed to evaluate the 50% Inhibitory Concentrations (IC_50_) values for compounds against ATG7 (autophagy-related protein-7), NAE (NEDD8-activating enzyme), UAE (ubiquitin activating enzyme) and SAE = SUMO-activating enzyme with appropriately tagged ubiquitin-like proteins and E2 conjugating enzymes as described in the Methods. GABARAP = GABAA receptor-associated protein. Data represent mean ± SEM. n = Number of independent experiments.

*v) Line 772 – Please provide a full legend for the Table contents as to what is each column value depicting.*

The Table legend has been expanded and now reads:

**Table 3. Activities of pyrazolopyrimidine sulfamates in selected biochemical assays.**

ATP consumption by *Pf*TyrRS (25 nM) in the presence of ATP (10 μM), tyrosine (200 μM), pyrophosphatase (1 unit/mL) and cognate tRNA^Tyr^ (4.8 μM), was measured using the Kinase Glo kits after incubation for 37°C for 1 h. Data are the average of three independent experiments and error bars correspond to SEM. The T_m_ values for *Pf*TyrRS and *Hs*TyrRS (2.3 μM) w measured in the apo form or after incubation at 37°C for 2 h with the nucleoside sulfamates (50 μM with *Pf*TyrRS and 200 μM with *Hs*TyrRS) in the presence of 10 μM ATP, 20 μM tyrosine, 4 μM cognate tRNA^Tyr^ (*Pf*TyrRS) or 8 mg/mL yeast tRNA (*Hs*TyrRS). *K_D_ values (apparent) are estimated from differential scanning fluorimetry (DSF) analysis using an irreversible protein thermal unfolding model that has been described previously (Hall, 2019). AMS = Adenosine 5'-sulfamate. n = Number of biological repeats. Data values represent mean ± SEM.

*vi) Please also move the description of the assay parameters to the DSF section in the methods.*

We have described the assay parameters in the Methods but prefer to also include them in the figure legend to make it easier for the reader. If the Editor prefers that we remove the parameters from the Figure Legend, we are willing to do so.

*vii) Possible to add a table in the supplementary tables mentioning the raw melt temperatures by virtue of which the delta Tms have been calculated?*

The raw melting temperatures have now been included in a new Table (Supplementary Table 12).

*Please add all the relevant graphs in the supplementary figures for the supplementary tables S1, S2, S3, S4 and S5.*

Relevant graphs for the supplementary tables S2, S3, S4 and S5 have now been included as Supplementary figures S2, S3, S4, S5. (Table S1 data was already plotted as Figure S1).

*Please refer to every graph, figure or table wherever a related point is referred to in the text. In some cases, it might get difficult for a reader to connect two points which might be obvious to specialists in the field.*

Each figure and table is now referred to in the text, at the relevant points.

Figure Files have been reviewed and uploaded.

Data Requirements:

All data are provided in the manuscript or Supplementary Information. The following structures have been deposited in the PDB: *Pf*TyrRS/Tyr-ML471 - PDB ID 9CLL

References have been reviewed.

-

Response References

Boddy, A., P. Edwards, and M. Rowland. 1989. Binding of sulfonamides to carbonic anhydrase: influence on distribution within blood and on pharmacokinetics. *Pharm Res*. 6:203-209.

Evans, M.E., W.C. Clark, G. Zheng, and T. Pan. 2017. Determination of tRNA aminoacylation levels by high-throughput sequencing. *Nucleic Acids Res*. 45:e133.

Hall, J. 2019. A simple model for determining affinity from irreversible thermal shifts. *Protein Sci*. 28:1880-1887.

Istvan, E.S., F. Guerra, M. Abraham, K.S. Huang, F. Rocamora, et al. 2023. Cytoplasmic isoleucyl tRNA synthetase as an attractive multistage antimalarial drug target. *Science translational medicine*. 15:eadc9249.

Kato, N., E. Comer, T. Sakata-Kato, A. Sharma, M. Sharma, et al. 2016. Diversity-oriented synthesis yields novel multistage antimalarial inhibitors. *Nature*. 538:344-349.

Supuran, C.T. 2010. Carbonic anhydrase inhibitors. *Bioorg Med Chem Lett*. 20:3467-3474.

Xie, S.C., R.D. Metcalfe, H. Mizutani, T. Puhalovich, E. Hanssen, et al. 2021. Design of proteasome inhibitors with oral efficacy in vivo against Plasmodium falciparum and selectivity over the human proteasome. *Proc Natl Acad Sci U S A*. 118:e2107213118.

Xie, S.C., Y. Wang, C.J. Morton, R.D. Metcalfe, C. Dogovski, et al. 2024. Reaction hijacking inhibition of *Plasmodium falciparum* asparagine tRNA synthetase. *Nat Commun*. 15:937.
